# A prospective, randomized, clinical trial comparing the hemodynamics, efficacy, and safety of 6% hydroxyethyl starch 130/0.4 compared to albumin in postoperative patients undergoing pancreaticoduodenectomy

**DOI:** 10.1186/cc10857

**Published:** 2012-03-20

**Authors:** SK Hong, K Kyoung, Y Kim, S Kim

**Affiliations:** 1 Ulsan University College of Medicine, Asan Medical Center, Seoul, South Korea; 2 Inje University College of Medicine, Harundae Paik Hospital, Busan, South Korea

## Introduction

Hypovolemia is often present in patients undergoing extensive abdominal surgery. As the first colloid used in the clinical setting, albumin is still widely employed during perioperative periods. We hypothesized that 6% hydroxyethyl starch (HES) 130/0.4 is equally efficacious and has the added advantages of its low cost and convenience of use. This study's objective is to compare the hemodynamics, efficacy, and safety of HES 130/0.4 compared with that of albumin.

## Methods

This study was a prospective, randomized, active-controlled study comparing the hemodynamics, efficacy, and safety of HES 130/0.4 to that of albumin in patients undergoing pancreaticoduodenectomy. Eligible adult patients of both sexes were assigned following the surgery into either the HES group or the albumin group at a ratio of 1:1. Crystalloids for hydration and colloid therapy for volume support were administered. The primary endpoint of this study was the hemodynamic evaluation. Secondary endpoints were measurement of the input-output, ICU stay, ventilation time, length of hospital stay, time to liquid mealtime and the use of blood products. Safety assessment was carried out by performing physical examination, laboratory examination, and assessment of any adverse events during the study period.

## Results

A total of 50 patients were randomized to study groups (25 each). The volume of the crystalloid was the same in both groups; however, significantly more colloids were infused after 24 hours post surgery in the HES group than in the albumin group, the voluven patient group had lower heart rates, and the difference in the lowest MAP value was -1.64 mmHg (lower limit of confidence interval, -8.228 mmHg) than in the albumin group. Routine hematology and biochemical profiles, including blood coagulation test and renal function assessment, were comparable in the two groups. The mean duration of the ICU stay, ventilation, hospital stay, and tolerance of a liquid meal were similar. The mean cost of the colloid was significantly lower in the HES 130/0.4 group than in the albumin group (*P *< 0.001).

## Conclusion

This study demonstrated that 6% HES 130/0.4 may be used as a valuable alternative to 5% albumin in patients undergoing extensive abdominal surgery, as its low cost is also of value.

